# Pulmonary artery wave reflection and right ventricular function after lung resection

**DOI:** 10.1016/j.bja.2022.07.052

**Published:** 2022-09-15

**Authors:** Adam Glass, Philip McCall, Alex Arthur, Kenneth Mangion, Ben Shelley

**Affiliations:** 1 Academic Unit of Anaesthesia, Pain and Critical Care, University of Glasgow, Glasgow, UK; 2 School of Anaesthesia, Northern Ireland Medical and Dental Training Agency, Belfast, UK; 3 Department of Anaesthesia, Golden Jubilee National Hospital, Clydebank, UK; 4 British Heart Foundation, Glasgow Cardiovascular Research Centre, University of Glasgow, Glasgow, UK

**Keywords:** afterload, cardiovascular magnetic resonance imaging, lobectomy, lung resection, right ventricle, strain, wave intensity analysis

## Abstract

**Background:**

Lung resection has been shown to impair right ventricular function. Although conventional measures of afterload do not change, surgical ligation of a pulmonary artery branch, as occurs during lobectomy, can create a unilateral proximal reflection site, increasing wave reflection (pulsatile component of afterload) and diverting blood flow through the contralateral pulmonary artery. We present a cardiovascular magnetic resonance imaging (MRI) observational cohort study of changes in wave reflection and right ventricular function after lung resection.

**Methods:**

Twenty-seven patients scheduled for open lobectomy for suspected lung cancer underwent cardiovascular MRI preoperatively, on postoperative Day 2, and at 2 months. Wave reflection was assessed in the left and right pulmonary arteries (operative and non-operative, as appropriate) by wave intensity analysis and calculation of wave reflection index. Pulmonary artery blood flow distribution was calculated as percentage of total blood flow travelling in the non-operative pulmonary artery. Right ventricular function was assessed by ejection fraction and strain analysis.

**Results:**

Operative pulmonary artery wave reflection increased from 4.3 (2.1–8.8) % preoperatively to 9.5 (4.9–14.9) % on postoperative Day 2 and 8.0 (2.3–11.7) % at 2 months (*P*<0.001) with an associated redistribution of blood flow towards the nonoperative pulmonary artery (r>0.523; *P*<0.010). On postoperative Day 2, impaired right ventricular ejection fraction was associated with increased operative pulmonary artery wave reflection (r=–0.480; *P*=0.028) and pulmonary artery blood flow redistribution (r=–0.545; *P*=0.011). At 2 months, impaired right ventricular ejection fraction and right ventricular strain were associated with pulmonary artery blood flow redistribution (r=–0.634, *P*=0.002; r=0.540, *P*=0.017).

**Conclusions:**

Pulsatile afterload increased after lung resection. The unilateral increase in operative pulmonary artery wave reflection resulted in redistribution of blood flow through the nonoperative pulmonary artery and was associated with right ventricular dysfunction.

**Clinical trial registration:**

NCT01892800.

**Editor's key points**

• Lung resection can impair right ventricular function, but pathophysiologic mechanisms remain poorly understood.
• Using cardiovascular MRI, the authors investigated changes in pulmonary artery wave reflection and right ventricular function in 27 patients undergoing open lobectomy for lung cancer.
• The unilateral increase in wave reflection in the pulmonary artery of the operated lung resulted in redistribution of blood flow to the nonoperative pulmonary artery, and was associated with right ventricular dysfunction.

Lung cancer is the leading cause of global cancer death,^1^ and whilst lung resection, in appropriate cases, offers the best chance of cure, it is associated with significant postoperative morbidity.^2^, ^3^, ^4^ After resection, impaired functional capacity^5^ appears to be influenced by a reduction in both respiratory and cardiac functions,^6^^,^^7^ with right ventricular (RV) dysfunction hypothesised to contribute to the impaired functional capacity.^6^^,^^8^ This RV dysfunction has historically (and intuitively) been attributed to an increase in RV afterload secondary to intraoperative one-lung ventilation, unilateral ligation of a proximal pulmonary artery (PA), and the removal of lung tissue. Previous studies have, however, consistently failed to demonstrate any persistent postoperative change in afterload, as assessed by pulmonary vascular resistance (PVR).^8^, ^9^, ^10^, ^11^, ^12^, ^13^, ^14^ Given the unilateral insult after lung resection, within this study, we aimed to investigate the changes in factors that oppose RV ejection in each individual lung and their contribution to RV dysfunction.

Complete assessment of afterload must include assessment of the resistance to both steady and pulsatile flows^15^^,^^16^ with the pulsatile components accounting for up to half of the hydraulic work of the RV.^17^^,^^18^ As PVR is calculated from the mean values of flow and pressure, it only assesses the resistance to steady flow across the entire pulmonary vasculature, ignoring pulsatile components.^17^ A major component of pulsatile afterload is wave reflection; this occurs at a vessel bifurcation (or a change in vessel calibre or compliance) and may significantly contribute to afterload.^15^ The site where wave reflection occurs can be determined by the timing of the returning reflected wave; for example, early wave reflection has been demonstrated in a PA branch (right PA [RPA] or left PA [LPA]) in the presence of a proximal pulmonary embolism.^19^ A proximal reflection site results in the return of the reflected wave to the ejecting RV in early to mid-systole, opposing forward flow and thus increasing RV wall stress and impairing RV function.^20^, ^21^, ^22^ The ligation of the lobar branch of the resected PA may create a similar proximal reflection site. Additionally, as lung resection causes a unilateral change in the pulmonary vasculature (in the operative PA), it might result in a unilateral increase in wave reflection, diverting blood flow through the unchanged contralateral vasculature (non-operative PA).

The reflective components of afterload can be modelled by wave intensity analysis (WIA), which allows assessment of wave travel and reflection in each individual PA.^23^ Originally described by Parker and Jones,^24^ WIA models forward and backward travelling waves from the resultant changes in blood flow and pressure that the waves cause. Forward compression waves (FCWs) are generated by ventricular contraction and travel away from the RV driving forward blood flow and pressure increase in systole. Backward compression waves (BCWs) are caused by reflection of the FCWs in the pulmonary vasculature and therefore travel back *towards* the RV, encouraging retrograde flow of blood, thus contributing to pulsatile afterload.^23^ Wave reflection can be quantified as wave reflection index (WRI), expressed as the percentage of the FCW that is reflected back towards the heart as a BCW.^25^

In this observational study, we tested the hypothesis that there is a unilateral increase in wave reflection after lung resection and that it is associated with impaired RV function. Secondly, we hypothesised that a unilateral increase in wave reflection results in a redistribution of blood flow through the contralateral PA, which may be a surrogate marker of afterload in this setting.

## Methods

We conducted a single-centre, prospective, observational cohort study in a tertiary referral cardiothoracic centre in Scotland. The study was prospectively registered on ClinicalTrials.gov (identifier: NCT1892800) in August 2013. Ethics approval was obtained from the West of Scotland Research Ethics Committee (134/WS/0055). Written informed consent was provided by all patients. Inclusion criteria were adult patients undergoing elective open lobectomy. Exclusion criteria included pregnancy, ongoing participation in investigational research that could undermine the scientific basis of the study, contraindication to cardiovascular magnetic resonance imaging (CMRI), video-assisted thoracoscopic surgery, or any planned resection other than lobectomy. Techniques were standardised for surgery (single surgeon and posterolateral muscle-sparing thoracotomy with lymph-node clearance, as appropriate) and anaesthesia (volatile anaesthetic, lung-protective ventilation, and thoracic epidural block).

### Cardiovascular magnetic resonance imaging

Cardiovascular magnetic resonance imaging (1.5 Tesla Siemens Avanto; Siemens, Erlangen, Germany) was performed preoperatively, on postoperative Day 2 (POD2), and at 2 months. ECG-gated fast-imaging steady-state free precession cines (TrueFISP; Siemens) were utilised throughout. Functional assessment slices of 6 mm thickness with a 4 mm gap between and voxel size 1.5 × 1.3 × 6 mm were measured. Short- and long-axis stacks of the ventricles were performed during breath holds. Flow analysis was performed in the pulmonary arteries (left and right), field of view 320–360 mm, slice thickness 5 mm, base resolution 256, phase resolution 100%, echo time 2.7 ms, repetition time 29.2 ms, and velocity encoding, as appropriate (150 cm s^−1^ for normal flow). Effective temporal resolution was one-thirtieth of the duration of the cardiac cycle.

**Fig 1:**
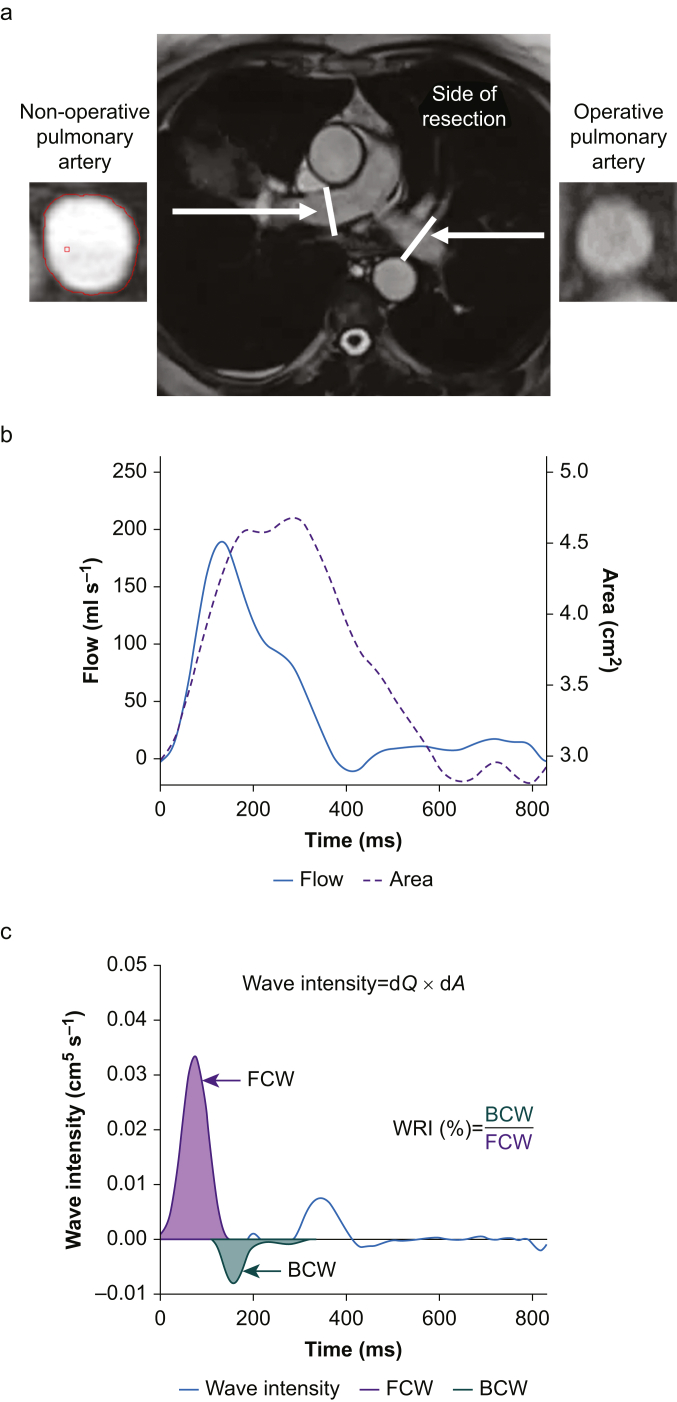
**Summary of wave intensity analysis methodology.** a. Cardiovascular MRI, central image display mapping scan for the left and right (operative and non-operative) pulmonary arteries with cross section of each artery displayed on either side. b. Flow *Q* and area *A* plots generated after analysis of pulmonary artery. c. Wave intensity (d*Q*×d*A*) is plotted by the blue line with the forward compression wave area (FCW; purple) and backward compression wave area (BCW; green) highlighted with calculation of wave reflection index (WRI) demonstrated. Indicative images from patient in study.

All scans were anonymised and randomised before reporting. Flow analysis was performed using proprietary software (Argus; Siemens, Erlangen, Germany) by two independent reporters.^26^ Tracing of the endocardial border of the branched PA was performed and interpolated throughout the cardiac cycle with visual inspection and augmentation performed for each phase. Flow (*Q*) and area (*A*) results for the 30 phases of the cardiac cycle were generated from the main PA (MPA), RPA, and LPA, termed operative or non-operative, as appropriate (Fig 1).

In patients with complete imaging at preoperative, POD2, and 2 months, strain analysis was performed on the cardiac four-chamber long-axis view using commercially available software (QStrain, version 2.1.12.2; Medis Medical Imaging, Leiden, The Netherlands).^27^ RV global longitudinal strain (RVGLS) and RV free-wall longitudinal strain (RVFWLS) were calculated for the RV. Left ventricular (LV) global longitudinal strain, LV global circumferential strain, and LV global radial strain were also calculated. Reporting and results of RV ejection fraction (RVEF) and LV ejection fraction (LVEF) have been described.^28^

Data processing and interpretation were performed, as detailed in R Studio Inc (Boston, MA, USA). The anonymised and randomised images were inspected, analysed, and interpreted before unblinding. To reduce signal noise, the dual reported flow and area results were averaged for each phase of the cardiac cycle, a 7-point second-order Savitzky–Golay filter^29^ was applied, and the resulting data were interpolated to 1 ms by a cubic spline without further data smoothing. Visual inspection of the flow and area plots was performed to assess for potential motion artifact. Total flow in each PA was calculated as the area under the flow *vs* time curve throughout the cardiac cycle. PA distribution of blood flow was assessed by the blood flow travelling in the non-operative PA divided by the sum of total blood flow in the operative and non-operative PAs. PA acceleration time (AT) was measured as the time to peak flow.^19^

Wave intensity analysis (WIA) was performed as per Quail and colleagues^19^ and is described in detail in Supplementary methods and depicted in Fig 1. Briefly, wave speed (also known as pulse wave velocity) was calculated by the ‘sum of squares’ method to minimise any influence of early wave reflection.^19^^,^^30^ The overall wave intensity throughout the cardiac cycle was calculated from the change in flow (d*Q*) multiplied by the change in area (d*A*) and separated into the underlying forward and backward component waves; area changes are commonly used as a surrogate marker for pressure changes for noninvasive WIA.^19^ An original R programme was written by AG to identify the individual waves in the forward and backward plots and calculate both the timing and magnitude of each wave. WRI was calculated as the area of the BCW relative to the area of the FCW, expressed as a percentage.^25^

### Statistical methods

All statistical analyses were performed in R Studio. Data are presented as mean (standard deviation) or median (inter-quartile range), as appropriate. Changes over time were assessed by one-way repeated measures analysis of variance or Friedman's test, as appropriate. *Post hoc* comparisons were performed with the paired *t*-test or Wilcoxon signed-rank test. Unpaired comparisons were performed by the unpaired *t*-test or Wilcoxon rank-sum test. Within-subject association testing between the change in two continual variables was assessed by performing analysis of covariance (ancova) with the patient as the factor, as described by Bland and Altman^31^ and interpreted as per Landis and Koch.^32^

## Results

Twenty-eight patients were recruited between September 2013 and September 2014, but one patient was discovered to have a ferromagnetic object within the chest wall on initial CMR imaging and excluded from further participation. CMR imaging was completed in 27 patients (100%) preoperatively, 22 patients (81.5%) on POD2, and 24 patients (88.9%) at 2 months. On POD2, three patients declined CMR imaging, one patient had a persistent air leak and CMR transfer was deemed unsafe, and one patient had an epidural catheter *in situ* that was not MR compatible.^33^ At 2 months, one patient declined, one patient was an inpatient at another hospital, and another patient had a contraindication to scanning.

The mean patient age was 67 (15.0) yr, and 17 (63%) were female. Twenty-two patients had a lobectomy, and four had a bilobectomy (including right middle lobe). One patient required on table conversion to pneumonectomy; the patient is included in the strain and WIA results although excluded for the PA blood flow distribution testing. Right-sided procedures were performed in 17 (63%) patients (Table 1 for all).

**Table 1:** Patient characteristics. Data expressed as number (%), mean (standard deviation), or median (inter-quartile range). COPD, chronic obstructive pulmonary disease; FEV_1_, forced expiratory volume in 1 s; FVC, forced vital capacity; IHD, ischaemic heart disease; PVD, peripheral vascular disease; SaO_2_, oxygen saturations; TLCO, transfer limit for carbon monoxide.

Patient characteristics	*n*=27
Age, yr	67.0 (15.0)
Female sex	17 (63%)
BMI, kg m^−2^	26.1 (3.9)
Preoperative pulmonary function	
SaO_2_ on air, %	96.4 (1.7)
FEV_1_, L	1.9 (1.6–2.4)
Percentage of predicted FEV_1_, %	87.5 (25.1)
FEV_1_/FVC, %	64.1 (14.8)
TLCO, ml kPa^−1^ min^−1^	5.2 (1.7)
Percentage of predicted TLCO, %	66.6 (15.2) TLCO
Comorbidities and risk score	
History of cancer	7 (25.9%)
Hypertension	9 (33.3%)
COPD	6 (22.2%)
IHD	6 (22.2%)
Diabetes mellitus	0
PVD	5 (18.5%)
Obesity	2 (7.4%)
Alcoholism	0
Thoracoscore	0.7 (0.3%)
Resection type	
Pneumonectomy	1 (3.7%)
Lobectomy	22 (81.5%)
Bilobectomy	4 (14.8%)
Segments resected	5 (3–5)
Right-sided procedure	17 (63%)
Pathology	
Primary lung cancer	24 (88.9%)
Other malignant	1 (3.7%)
Benign disease	2 (7.4%)
Smoking status	
Current smoker	13 (46.4%)
Ex-smoker	12 (42.9%)
Never smoked	2 (7.1%)
Pack years	38.2 (21.7)

### Ventricular function

Heart rate and cardiac output were increased on POD2 but returned to baseline at 2 months (Table 2). Twenty patients had complete imaging at all three time points and were included in strain analysis. RV strain analysis was possible in 58 of 60 (96.7%) scans. Two scans had a marked non-physiological increase in RV free-wall length in early systole considered secondary to motion artifact and were excluded from analysis before unblinding. RVGLS and RVFWLS were unchanged on POD2 but reduced (i.e. became less negative, indicating deteriorating RV function) at 2 months (*P*=0.022 and *P*=0.023, respectively; paired *t*-tests). LV strain was unchanged throughout the study.

**Table 2:** Ventricular function and pulmonary artery blood flow. Values are mean (standard deviation). EDV, end diastolic volume; EF, ejection fraction; ESV, end systolic volume; FWLS, free-wall longitudinal strain; GCS, global circumferential strain; GLS, global longitudinal strain; GRS, global radial strain; LV, left ventricle/ventricular; MPA, main pulmonary artery; PA, pulmonary artery; POD2, postoperative Day 2; RV, right ventricle/ventricular; SV, stroke volume. ∗One-way repeated measures analysis of variance. ^†^Significant difference from preoperative. ^‡^Significant difference from POD2. Both paired *t*-test. ^¶^Significant difference to non-operative PA; unpaired *t*-test. Significant results (P<0.05) are highlighted in italic. RVEF and LVEF and volume results published previously.^28^

Parameter	Preoperative	POD2	2 months	*P*-value∗
Heart rate (beats min^−1^)	64.4 (13.0)	77.0 (11.0)^†^	69.4 (10.3)^‡^	*0.002*
Cardiac output (L min^−1^)	5.73 (1.63)	6.91 (1.62)^†^	6.12 (1.64)^‡^	*0.002*
RVEF (%)	50.5 (6.9)	45.6 (4.5)^†^	44.9 (7.7)^†^	*0.003*
RVEDV (ml)	119.1 (25.4)	125.9 (22.5)	109.4 (31.6)^†‡^	*0.019*
RVESV (ml)	59.8 (17.1)	68.6 (14.5)^†^	59.8 (17.6)^‡^	*0.040*
RVSV (ml)	59.3 (12.0)	57.3 (10.7)	49.6 (16.5)^†^	*0.002*
LVEF (%)	58.4 (7.1)	57.4 (7.3)	59.7 (9.3)	0.621
LVEDV (ml)	109.2 (19.5)	106.3 (19.2)	93.6 (28.2)^†‡^	*0.001*
LVESV (ml)	46.0 (13.2)	46.0 (14.2)	37.7 (13.1)^†‡^	*0.019*
LVSV (ml)	63.2 (11.7)	60.3 (9.0)	55.9 (18.0)^†^	*0.004*
RVGLS (%)	–32.7 (6.2)	–30.1 (6.3)	–28.1 (7.3)^†^	*0.006*
RVFWLS (%)	–37.3 (10.2)	–36.5 (9.1)	–32.5 (10.6)^†^	*0.025*
LVGLS (%)	–25.4 (5.0)	–26.2 (3.4)	–25.3 (4.4)	0.739
LVGCS (%)	–33.3 (6.5)	–34.7 (6.6)	–33.0 (7.7)	0.500
LVGRS (%)	70.7 (18.5)	74.0 (17.7)	69.2 (19.7)	0.524
MPA acceleration time (ms)	116 (21)	83 (19)^†^	104 (20)^†‡^	*<0.001*
Proportion of blood flow (%)				
Non-operative PA	48.10 (6.20)	66.28 (9.53)^†^	60.84 (11.68)^†‡^	*<0.001*
Operative PA	51.90 (6.20)	33.72 (9.53)^†¶^	39.16 (11.68)^†‡¶^	*<0.001*

### Pulmonary artery blood flow and wave intensity analysis

**Fig 2:**
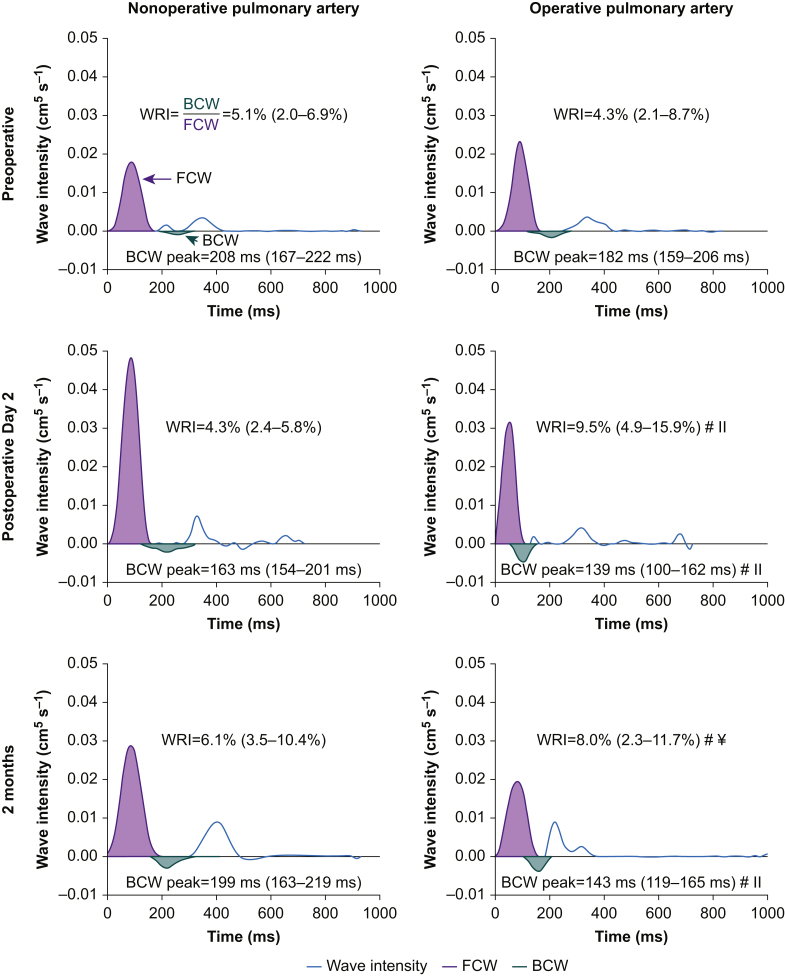
**Representative example of the wave intensity analysis changes after lung resection**. Wave intensity analysis plots are displayed for the nonoperative (left column) and operative (right column) PAs, preoperatively (top row), on postoperative Day 2 (POD2; middle row), and at 2 months (bottom row). Net wave intensity is plotted by the blue line with the forward compression wave area (FCW; purple) and backward compression wave area (BCW; green) highlighted. Wave reflection index (WRI) and time to peak BCW results from the entire study population are noted on each plot. ^#^Significant difference from preoperative, ^¥^Significant difference from POD2; both Wilcoxon signed-rank test. ^ll^Significant difference from non-operative PA at same time point; Wilcoxon rank-sum test.

In total, flow/area plots were available for 215 vessels (73 MPAs, 70 LPAs, and 72 RPAs). Visual inspection of MPA area plots, however, revealed movement artifact in the majority of scans preventing WIA in the MPA. Inspection of operative and non-operative PA WIA plots revealed abnormalities in six scans (4.2%); all had marked late diastolic increases in area without corresponding changes in flow. This was considered not to be a true physiological change but secondary to motion artifact, as observed previously in the MPA,^19^^,^^34^ two scans were preoperative, one was on POD2, and three were at 2 months. These six scans were removed from analysis before unblinding, leaving 68 operative and 68 non-operative PAs (71 RPA and 65 LPAs). The changes in WIA are shown in Fig 2 and Supplementary Table 2.

There was a marked postoperative redistribution of blood flow with an increased percentage travelling through the non-operative PA compared with the operative PA (*P*<0.001; paired *t*-test; Table 2). The flow percentage changes for the right- and left-sided resections are presented in Supplementary Table 3. WRI was increased in the operative PA on POD2 and at 2 months (*P*=0.001 and *P*=0.026, respectively; Wilcoxon signed rank) compared with preoperative. Although the BCW area increased in the non-operative PA postoperatively, the FCW area increased in a similar ratio; therefore, non-operative PA WRI was unchanged throughout the study.

The time to peak BCW was reduced in the operative PA (*P*<0.005; Wilcoxon signed rank) and was less than the non-operative PA at both postoperative time points (*P*=0.040 and *P*=0.003; Wilcoxon rank sum; Fig 2). There were moderate-to-strong postoperative within-subject associations between the change in PA blood flow distribution from preoperative and both the change in operative PA WRI (*r*=0.728, *P*<0.001 on POD2; *r*=0.523, *P*=0.010 at 2 months) and the change in time to peak BCW in the operative PA (*r*=–0.653, *P*=0.002 on POD2; *r*=–0.594, *P*=0.006 at 2 months), all within-subject ancova with patient as a factor.

### Associations between wave reflection, blood flow distribution, and right ventricular function

On POD2, there were moderate negative within-subject associations between the change in RVEF (from preoperative to POD2) and both changes in operative PA WRI and PA blood flow distribution from preoperative (Table 3). At 2 months, the within-subject change in PA blood flow distribution was strongly associated with reduction in RVEF and moderately associated with changes in both RVGLS and RVFWLS. The reduction in time to peak BCW in the operative PA was associated with reduction in RVEF on POD2 (*r*=0.505; *P*=0.024) and at 2 months (*r*=0.509; *P*=0.026), both within-subject ancova with patient as a factor. A within-subject increase in non-operative PA WRI was strongly associated with a reduction in RVGLS and RVFWLS at 2 months. In each association, increased WRI or redistribution of PA blood flow through the non-operative PA was associated with an impairment in RV function.

**Table 3:** Within-subject association between right ventricular function and measures of afterload. POD2, postoperative Day 2; RVEF, right ventricular ejection fraction; RVFWLS, right ventricular free-wall longitudinal strain; RVGLS, right ventricular global longitudinal strain; WRI, wave reflection index. Analysis of covariance with patient as a factor. Significant results (P<0.05) are highlighted in *italic*.

Parameter	Pulmonary artery		RVEF (%)	RVGLS (%)	RVFWLS (%)
Change between preoperative and POD2					
WRI (%)	Operative	*r*	*–0.480*	0.015	–0.174
		*P*	*0.028*	0.952	0.518
	Non-operative	*r*	–0.024	0.337	0.311
		*P*	0.920	0.186	0.124
Blood flow distribution (%)	Non-operative	*r*	*–0.545*	0.313	–0.087
		*P*	*0.011*	0.237	0.748
Change between preoperative and 2 months					
WRI (%)	Operative	*r*	–0.070	0.450	*0.487*
		*P*	0.763	0.053	*0.035*
	Non-operative	*r*	–0.268	*0.702*	*0.745*
		*P*	0.240	*0.001*	*<0.001*
Blood flow distribution (%)	Non-operative	*r*	*–0.634*	*0.471*	*0.540*
		*P*	*0.002*	*0.042*	*0.017*

## Discussion

Pulsatile afterload (PA wave reflection) was increased after lung resection, which was associated with impaired RV function. Assessment of each individual PA revealed a unilateral increase in wave reflection in the operative PA and a redistribution of blood flow through the contralateral PA that are associated with impaired RV function. Although RV strain was initially maintained, impaired RV strain occurred by 2 months postoperatively.

Lung resection is a unilateral insult to the pulmonary vasculature; as such, conventional approaches to measure RV afterload across the entire pulmonary circulation may not detect important changes. Previous authors have demonstrated that PVR and PA pressures are unchanged at rest after lung resection, with no association demonstrated between changes in afterload (as measured by PVR) and RV function.^8^, ^9^, ^10^, ^11^, ^12^, ^13^, ^14^ As PVR predominantly assesses the resistance to steady flow in the distal pulmonary vasculature, it will fail to detect proximal changes in the pulsatile components of afterload.^15^^,^^35^ Ligation of the lobar branch of the PA during lung resection may result in the creation of a proximal site of wave reflection and predominately change the *pulsatile* components of afterload without any change in PVR. At normal PA pressure, as demonstrated previously after lung resection,^8^^,^^12^, ^13^, ^14^ the pulsatile components of afterload account for a greater percentage of total RV afterload (up to 50%) than at higher PA pressures.^15^

The wave intensity analysis models afterload in the time domain and can be performed in any vessel. This allows assessment of wave reflection in each individual lung and allows the changes observed to be temporally related to the cardiac cycle.^23^ To interpret the impact of wave reflection on RV afterload, it must be assessed in the proximal MPA. In our study, WIA could not be performed in the MPA, as the area measurements were subject to significant motion artifact, similar to previous studies.^19^^,^^34^ Analysis of the MPA flow profiles, however, allows assessment of a surrogate marker of wave reflection; increased wave reflection causes a ‘notching’ of the PA flow profile and a reduction in AT.^19^ The reduction in AT in the MPA suggests that the operative PA wave reflection caused increased wave reflection in the MPA. Between preoperative and POD2, the within-subject change in AT in the MPA was strongly associated with the changes in operative PA WRI (*r*=–0.663; *P*=0.001), PA blood flow redistribution (*r*=–0.621; *P*=0.003), and RVEF (*r*=0.571; *P*=0.004), all within-subject ancova with patient as a factor. None of these associations were significant between the preoperative and 2 month time points.

In the operative PA, the postoperative increase in WRI and reduction in time to peak BCW are in keeping with wave reflection occurring from a more proximal site than preoperatively. This is supported by calculation of the apparent distance to reflection (see Supplementary methods),^19^ which is reduced from 6.9 (5.0–9.2) cm preoperatively to 2.6 (2.0–4.7) cm on POD2 and 3.2 (2.2–5.2) cm at 2 months (*P*<0.001; Wilcoxon signed rank for both). The origin of the new reflection site in the operative PA may therefore be the anatomical site of surgical ligation of the lobar PA. Similar to pulmonary hypertension, the arrival of a BCW from a proximal reflection site earlier in systole appears to impair RV function.^20^^,^^21^ The increase in operative PA WRI is lower than observed in pulmonary hypertension (9.5 [4.9–14.9]% on POD2 in this study *vs* 20–31.8% reported in pulmonary hypertension).^19^^,^^36^ Additionally, there is only a moderate association between WRI and RVEF on POD2 with no association at 2 months. The consistent associations between the changes in both the magnitude and timing of operative PA wave reflection and the changes in PA blood flow distribution suggest that unilateral wave reflection drives redistribution of blood flow. In cases where capacity exists, the non-operative lung may compensate for the contralateral increase in wave reflection and mask the true increase. This may explain why there are more consistent associations between impaired RV function and PA blood flow redistribution than operative PA WRI. In the setting of a unilateral increase in wave reflection, the PA blood flow distribution may be a surrogate marker of afterload (i.e. where the greater the proportion of blood flowing through the non-operative lung, the greater the afterload).

In this study, there is evidence of both acute and chronic postoperative increases in wave reflection associated with impaired RV function. On POD2, the acute changes in operative PA WRI and PA blood flow distribution are associated with impairment of RVEF. At 2 months, the change in PA blood flow distribution is associated with impaired RVEF, RVGLS, and RVFWLS. The increases in WRI are associated with impaired RVGLS and RVFWLS at 2 months; interestingly, the association is strongest with the change in non-operative PA WRI, suggesting that if the non-operative PA is unable to accommodate the increased blood flow (i.e. no capacity to compensate), then impaired RV strain may develop over time.

The results of this study highlight the potential pathophysiological mechanism by which RV dysfunction occurs after lung resection. At rest, the non-operative lung may initially be able to compensate for increased wave reflection in the contralateral lung; however, this may be insufficient with increasing cardiac output. Previous studies investigating the response to exercise after lung resection have demonstrated a rapid postoperative increase in PVR as cardiac output increases^8^^,^^13^^,^^14^ with further impairment of RV function.^12^ Similarly, this compensatory mechanism may be insufficient in the setting of critical illness; in a recent retrospective cohort study, the presence of ‘RV dysfunction’ was independently associated with mortality in unplanned admission to intensive care after lung resection.^37^ Further studies should assess the changes in RV function and afterload in response to stress after lung resection.

The potential limitations of the study include the temporal resolution of CMR imaging, the use of PA area as a surrogate of PA pressure, and the lack of a load-independent measure of RV function. Wave reflection generates a short-lived BCW, which a low temporal resolution CMR scan may underestimate or fail to detect. High CMR temporal resolution requires long breath holds or prolonged free-breathing sequences to limit motion artifact.^38^ Patients undergoing lung resection would likely be unable to complete this, and it may significantly prolong the scanning process. The design of a noninvasive CMR study and imaging protocol was a pragmatic balance between data acquisition and patient experience. Additionally, an ideal measure of RV contractility would be load independent.^39^ RV strain and RVEF are both influenced by loading conditions, in particular afterload,^39^^,^^40^ and the reductions demonstrated may therefore be influenced by increased afterload. This may be overcome by assessing RV function over varying loading conditions, such as on exercise^15^ or by invasive assessment of RV pressure–volume loops.^41^

Further investigation is required to determine if the changes in RV function and afterload demonstrated in this study impact patient-centred outcomes. Having now confirmed the existence of the association between wave reflection and RV function, this now paves the way for testing of novel therapeutic strategies aimed at aiding adaptation to the unique perioperative physiology seen in patients undergoing thoracic surgery with a view to preventing cardiovascular complications and improving long-term functional capacity.^42^

## Conclusions

Pulsatile afterload was increased after lung resection. The unilateral increase in operative PA wave reflection resulted in redistribution of blood flow through the non-operative PA and was associated with RV dysfunction.

## Authors' contributions

Study conception: BS.

Obtaining of funds: BS.

Supervising all aspects of the study: BS.

Patient recruitment: PM, AA.

Data acquisition: PM, AA.

Data reporting/analysis/interpretation: AG.

Dual reporting of cardiovascular magnetic resonance images: AA.

Dual reporting of strain analysis: KM.

Drafting of final manuscript: AG.

Critical revision of manuscript: AG, PM, BS.

Approval of final manuscript: all authors.
