# Supplementary material for: Pulmonary artery wave reflection and right ventricular function after lung resection
Source: Br J Anaesth. 2022 Sep 15;130(1):e128–36. doi: 10.1016/j.bja.2022.07.052 (PMC9875909; doi:10.1016/j.bja.2022.07.052)
Supplement: Multimedia component 1 [file mmc1.docx]

**Supplementary**

**Methods**

Wave speed (c) was calculated by the “Sum of Squares” method, Equation 1. This technique was originally devised in coronary arteries to calculate wave speed by minimising the influence of early wave reflection^1, 2^.

Separation of the Q, A and dI plots into their forward (+) and backward (-) components was performed as per Quail and colleagues^1^, Equation 3, Equation 4 and Equation 5. Timing of the waveform was referenced to the onset of the initial increase in dI to remove any discrepancy in the timing of waves secondary to differing distances of the imaging plane from the pulmonary valve. An increased distance would artificially prolong the arrival of waves due to the temporal delay in their arrival^2^, Figure 1.

In order to assess for motion artefact, visual inspection of the dI plots was performed prior to unblinding. Motion artefact is common in the MPA^3^. The time to peak and area under the curve of each wave were calculated using an original R Studio programme. The initial FCW and subsequent forward expansion wave (FEW) were detected from the dI+ plot with the nature of each wave confirmed by assessing the dQ+ at the peak of the waveform. The predominant backward wave from the dI- plot occurring between the peaks in FCW and FEW was detected to ensure identification of the wave generated by reflection of the FCW. The backward wave was determined to be either a BCW if the dQ was negative or a backward expansion wave (BEW) if dQ was positive. WRI was calculated as the ratio of the area of the BCW to the area of the FCW, if the backward wave was a BEW WRI was calculated as 0, Equation 6.

The distance to the apparent site of reflection was calculated by half the time difference between the peak of the BCW and the peak of the FCW multiplied by c^1^, Equation 7.

Table 1 Equations

| 1 | Wave speed (c) | $\boldsymbol{c =}\sqrt{\frac{\sum\mathbf{dQ}^{\boldsymbol{2}}}{\sum\mathbf{dA}^{\boldsymbol{2}}}}$ |
| --- | --- | --- |
| 2 | Net wave intensity (dI) | $dI=dQ.dA$ |
| 3 | Flow wave separation | ${dQ}_{\pm}= \frac{1}{2}(dQ \pm cdA)$ |
| 4 | Area wave separation | ${dA}_{\pm}= \frac{1}{2}(dA \pm\frac{1}{c}dQ)$ |
| 5 | Wave intensity separation | ${dI}_{\pm}= \pm\frac{c}{4}\left[ dA \pm\frac{dQ}{c} \right]^{2}$ |
| 6 | Wave reflection index (WRI) | $WRI = \frac{\sum BCW}{\sum FCW}$ |
| 7 | Distance to reflection site | $= \frac{c}{2}\left( Time to peak BCW-Time to peak FCW \right)$ |

c= Wave speed. Q= flow. A= Area. I= wave intensity. BCW= backward compression wave. FCW= forward compression wave. WRI= wave reflection index.


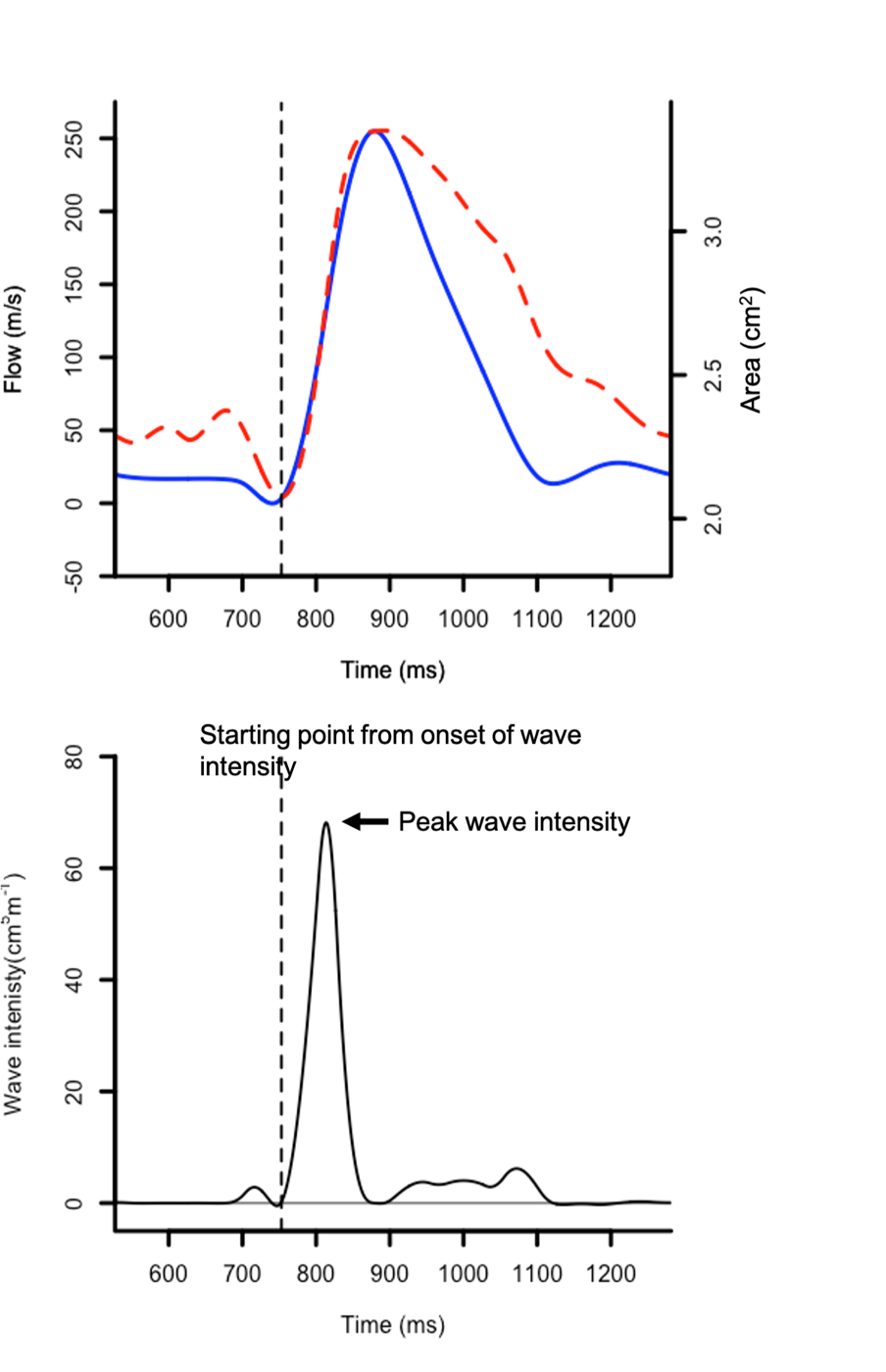


**Figure 1** **Example of net wave intensity calculation and detection of starting point**

Top plot, flow (blue solid line) and area (red dashed line) against time from a loop of the cardiac cycle. Starting time point of the plot is after 70% of the preceding cardiac cycle. Bottom plot, net wave intensity (dI) against time. Note rapid increase in dI coincides with the increase in flow and area of top plot. Peak wave intensity measured as the maximal value of dI. The starting point is the latest time preceding this peak at which dI=0 or there is a turning point in dI (measured by the time at which the temporal derivative of dI=0) prior to this peak. Indicative images from patient in study.

Supplementary results

Table 2 Wave intensity analysis results

|  | Operative pulmonary artery | | | | Non-operative pulmonary artery | | | |
| --- | --- | --- | --- | --- | --- | --- | --- | --- |
| Parameters | Pre-op | POD2 | Two months | P-value | Pre-op | POD2 | Two months | P-value |
|  | n=27 | n=20 | n=21 |  | n=25 | n=21 | n=22 |  |
| Blood flow (l/min) | 2.95 (0.81) | 2.29 (0.73) # | 2.33 (0.75) # | ***<0.001 **** | 2.78 (0.95) | 4.68 (1.42) # ll | 3.84 (1.45) # ¥ ll | ***<0.001 **** |
| Wave speed (m.s^-1^) | 1.38 (1.28-1.53) | 0.92 (0.73-1.20) # | 0.90 (0.75-1.06) # | ***<0.001 §*** | 1.29 (1.06-1.76) | 1.60 (1.33-1.97) # ll | 1.27 (1.10-1.78) ¥ ll | ***0.019 §*** |
| Acceleration time (ms) | 124.7 (19.3) | 82.1 (23.0) # | 106.0 (23.9) # ¥ | ***<0.001 **** | 128.0 (24.8) | 100.5 (18.8) # ll | 121.4 (20.4) ¥ ll | ***<0.001 **** |
|  |  |  |  |  |  |  |  |  |
| Forward compression wave | | | | | | | | |
| Area (x10^-3^ cm^5^) | 1.54 (1.14-2.20) | 1.38 (0.55-1.71) # | 1.15 (0.72-1.57) # | ***0.001 §*** | 1.35 (1.01-2.51) | 2.55 (1.94-3.38) # ll | 2.27 (1.56-3.73) # ¥ ll | ***0.001 §*** |
| Time to peak (ms) | 77 (19) | 63 (17) # | 70 (22) ¥ | ***0.022 **** | 86 (24) | 73 (17) # | 77 (17) | ***0.017 **** |
|  |  |  |  |  |  |  |  |  |
| Backward compression wave | | | | | | | | |
| Number of BCW (n/total) | 26/27 | 20/20 | 20/21 | 0.607 † | 23/25 | 20/21 | 20/22 | 0.223 † |
| Area (x10^-3^ cm^5^) | 0.08 (0.04-0.13) | 0.14 (0.04-0.23) # | 0.08 (0.02-0.13) ¥ | ***0.025 §*** | 0.07 (0.03-0.12) | 0.11 (0.06-0.24) # | 0.14 (0.05-0.27) # | ***0.003 §*** |
| Time to peak (ms) | 182 (159-206) | 140 (100-162) # | 143 (119-165) # | ***0.010 §*** | 208 (167-222) | 163 (154-201) ll | 199 (163-219) ll | 0.204 *§* |
|  |  |  |  |  |  |  |  |  |
| Wave reflection index (%) | 4.3 (2.1-8.7) | 9.5 (4.9-14.9) # | 8.0 (2.3-11.7) # ¥ | ***<0.001 §*** | 5.1 (2.0-6.9) | 4.3 (2.4-5.8) ll | 6.1 (3.5-10.4) | 0.197 § |
| Distance to reflection site (cm) | 6.9 (5.0-9.3) | 2.6 (2.0-4.7) # | 3.2 (2.2-5.2) # | ***0.001* §** | 6.9 (4.9-11.2) | 8.5 (4.6-10.0) ll | 7.8 (5.9-10.0) ll | 0.663 § |

Values are mean (SD) or median (IQR). POD2 = post-operative day 2. BCW= backward compression wave. *= one-way repeated measures ANOVA. *§*= Friedman’s test. Comparison between time-points paired t-test or Wilcoxon signed rank test. #= significant difference from pre-op. ¥= significant difference from post-operative day 2 (POD2). ll Significant difference from operative PA. †= Cochran test. Significant results (p<0.05) highlighted ***bold italics***

Table 3 Comparison of left vs right sided resections

| Proportion of blood flow (%) | | Pre-op | | POD2 | | Two months | | p-value |
| --- | --- | --- | --- | --- | --- | --- | --- | --- |
| All resections | | | | | | |  |  |
| Non-operative PA | 48.10 (6.20) | | 66.28 (9.53) # ll | | 60.84 (11.68) # ¥ ll | | ***<0.001*** * | |
| Operative PA | 51.90 (6.20) | | 33.72 (9.53) # | | 39.16 (11.68) # ¥ | | ***<0.001*** * | |
|  |  | |  | |  | |  | |
| Right sided resections |  | |  | |  | |  | |
| Non-operative PA / left PA | 45.68 (4.83) | | 64.55 (11.00) # ll | | 59.52 (12.81) # ll | | ***<0.001*** *§* | |
| **Operative PA / right PA** | 54.32 (4.83) | | 35.45 (11.00) # | | 40.48 (12.81) # | | ***<0.001*** *§* | |
|  |  | |  | |  | |  | |
| Left sided resections |  | |  | |  | |  | |
| **Non-operative / right PA** | 52.21 (6.30) | | 68.88 (6.68) # ll | | 64.38 (7.74) # ll | | ***0.006*** *§* | |
| Operative PA / left PA | 47.79 (6.30) | | 31.12 (6.68) # | | 35.62 (7.74) # | | ***0.006*** *§* | |

PA = pulmonary artery. POD2 = post-operative day 2. *= one-way repeated measures ANOVA. *§* = Friedman’s test. Comparison between time-points paired t-test or Wilcoxon signed rank test. # = significant difference from pre-op. ¥ = significant difference from post-operative day 2 (POD2). ll Significant difference from operative PA.

References

1 Quail MA, Knight DS, Steeden JA, et al. Noninvasive pulmonary artery wave intensity analysis in pulmonary hypertension. *Am J Physiol Heart Circ Physiol* 2015; **308**: H1603-11

2 Weir-McCall JR, Kamalasanan A, Cassidy DB, Struthers AD, Lipworth BJ, Houston JG. Assessment of proximal pulmonary arterial stiffness using magnetic resonance imaging: effects of technique, age and exercise. *BMJ Open Respir Res* 2016; **3**: e000149

3 Laffon E, Bernard V, Montaudon M, Marthan R, Barat JL, Laurent F. Tuning of pulmonary arterial circulation evidenced by MR phase mapping in healthy volunteers. *J Appl Physiol (1985)* 2001; **90**: 469-74
